# Flying and "Air Sickness."

**Published:** 1920-01-17

**Authors:** 


### FLYING AND "AIR SICKNESS."

AN interesting lecture was delivered recently at a meeting of the Royal Aeronautical Society by Dr. C. A. Swan, followed by a discussion.

Dr. Swan emphasised the importance of the subject in view of the rapid approach of commercial aviation and increasing passenger transport. He purposely adopted the popular rather than the purely scientific standpoint, and gave an account of his personal experiences in the rarefied atmosphere of mountain altitudes. The physical and psychical factors appear to be considerably intermingled: the material effects of a low atmospheric pressure are frequently accentuated by emotional disturbances. It seems that there is a definite altitude at which each individual's compensating mechanism begins to feel the strain; and individual capacity for readjustment is of greater importance than moderate changes in the environment. Muscular effort naturally increases symptoms of distress, which are variously exhibited as dyspnœa, cyanosis, vertigo, headache, tinnitus, vomiting, fainting, mental excitement, etc.

Simple remedies, such as black coffee, salol, and the old guide's remedy of oil of cinnamon on sugar, frequently effect relief, whilst the influence of posture is usually marked. The digestive organs and the higher nerve centres play a very large part.

Two types of altitude sickness have been described by Dr. Ravenhill, an acute form and a slow form, in which compensation is gradually lost. In the latter, cardiac or nervous symptoms may predominate. Compensation is attained by rise of blood pressure and by hyperpnœa, both of which may be ascribed to a response of the medullary centres to lack of oxygen and psychical influences. In acclimatisation the acidity of the blood due to non-volatile acids is increased, so that the respiratory centre becomes properly excited, although the alveolar carbon dioxide tension is low; in those who habitually dwell in mountain altitudes the red blood corpuscles are actually increased.

Dr. Swan observed that "staleness" in pilots is essentially due to fatigue of the mechanism for acclimatisation, and that rest is the first obvious point in treatment. Other causes of fatigue, such as eye-strain, dental sepsis, blocked nostrils, and mental worry should be eliminated as far as possible. But the pilot flying under war conditions, driving a fast-climbing machine and repeatedly encountering alarming evidence of "hate," presents a very different problem from those who fly in civil life, although a remarkable proportion of war pilots came to grief through influence of altitude, whilst many flew successfully who, by all the rules of the game, should have been unfit for aviation in any circumstances.

In the discussion following Dr. Swan's address, General Brooke Popham laid stress on the importance of oxygen administration, and said that great benefit was derived from its use in France during the war. Dr. Stamm considered that the question of air-sickness will affect airship rather than aeroplane flying, and that the provision of oxygen affords no real difficulty. The chairman, Brigadier-General Bagnall Wild, attaches importance to a healthy digestion, and expressed the opinion that there need be no fear of suffering during passenger flights, apart from excessive altitudes (over 15,000 feet) and "stunting."

Aviation as a means of transport would appear to present no serious terrors in this direction to the healthy individual. Our systems are far more adaptable than we are too often encouraged to believe.

